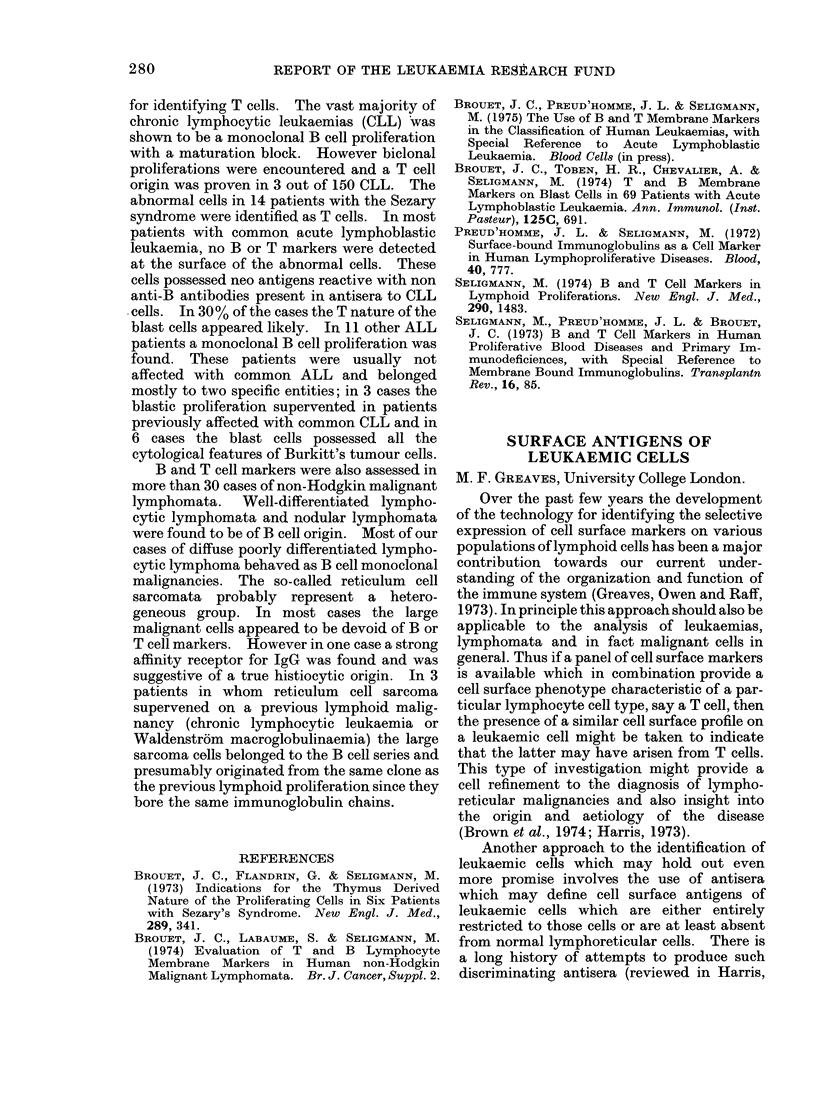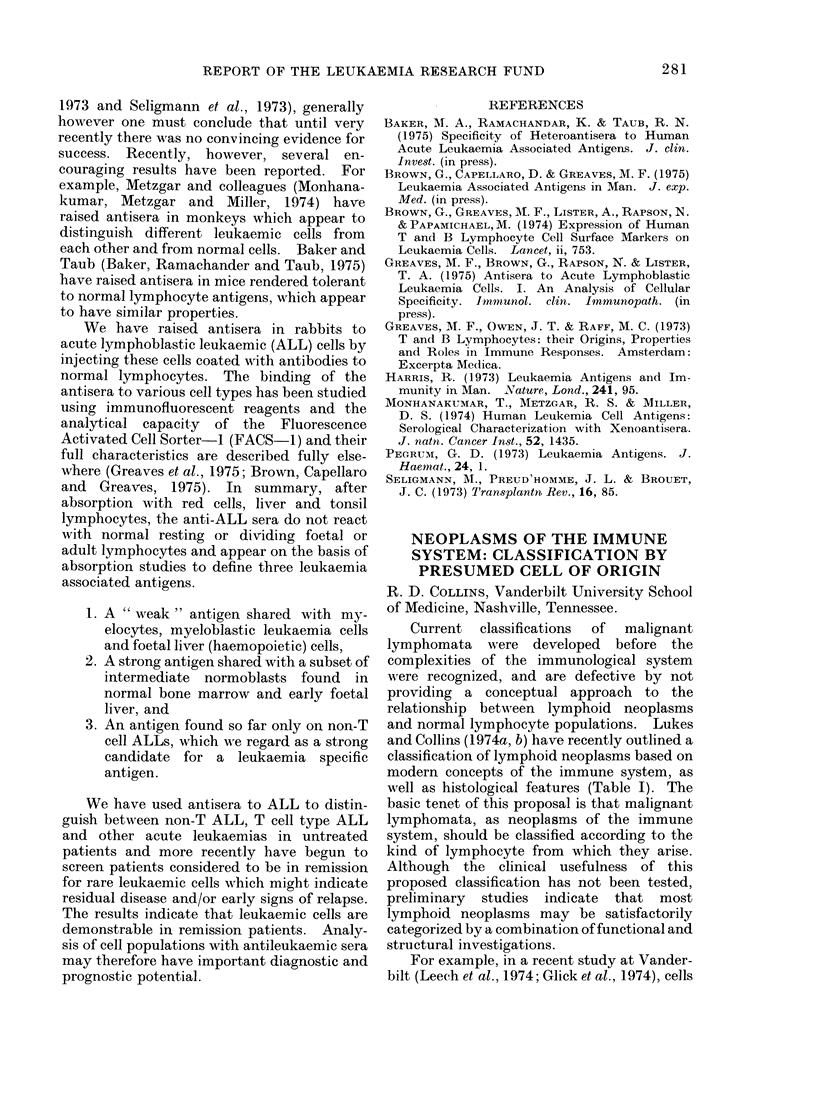# Proceedings: Surface antigens of leukaemic cells.

**DOI:** 10.1038/bjc.1975.218

**Published:** 1975-08

**Authors:** M. F. Greaves


### SURFACE ANTIGENS OF LEUKAEMIC CELLS

M. F. Greaves, University College London.

Over the past few years the development of the technology for identifying the selective expression of cell surface markers on various populations of lymphoid cells has been a major contribution towards our current understanding of the organization and function of the immune system (Greaves, Owen and Raff, 1973). In principle this approach should also be applicable to the analysis of leukaemias, lymphomata and in fact malignant cells in general. Thus if a panel of cell surface markers is available which in combination provide a cell surface phenotype characteristic of a particular lymphocyte cell type, say a T cell, then the presence of a similar cell surface profile on a leukaemic cell might be taken to indicate that the latter may have arisen from T cells. This type of investigation might provide a cell refinement to the diagnosis of lymphoreticular malignancies and also insight into the origin and aetiology of the disease (Brown *et al.*, 1974; Harris, 1973).

Another approach to the identification of leukaemic cells which may hold out even more promise involves the use of antisera which may define cell surface antigens of leukaemic cells which are either entirely restricted to those cells or are at least absent from normal lymphoreticular cells. There is a long history of attempts to produce such discriminating antisera (reviewed in Harris,

1973 and Seligmann *et al.*, 1973), generally however one must conclude that until very recently there was no convincing evidence for success. Recently, however, several encouraging results have been reported. For example, Metzgar and colleagues (Monhanakumar, Metzgar and Miller, 1974) have raised antisera in monkeys which appear to distinguish different leukaemic cells from each other and from normal cells. Baker and Taub (Baker, Ramachander and Taub, 1975) have raised antisera in mice rendered tolerant to normal lymphocyte antigens, which appear to have similar properties.

We have raised antisera in rabbits to acute lymphoblastic leukaemic (ALL) cells by injecting these cells coated with antibodies to normal lymphocytes. The binding of the antisera to various cell types has been studied using immunofluorescent reagents and the analytical capacity of the Fluorescence Activated Cell Sorter—1 (FACS—1) and their full characteristics are described fully elsewhere (Greaves *et al.*, 1975; Brown, Capellaro and Greaves, 1975). In summary, after absorption with red cells, liver and tonsil lymphocytes, the anti-ALL sera do not react with normal resting or dividing foetal or adult lymphocytes and appear on the basis of absorption studies to define three leukaemia associated antigens.

1. A " weak " antigen shared with myelocytes, myeloblastic leukaemia cells and foetal liver (haemopoietic) cells,
2. A strong antigen shared with a subset of intermediate normoblasts found in normal bone marrow and early foetal liver, and
3. An antigen found so far only on non-T cell ALLs, which we regard as a strong candidate for a leukaemia specific antigen.

We have used antisera to ALL to distinguish between non-T ALL, T cell type ALL and other acute leukaemias in untreated patients and more recently have begun to screen patients considered to be in remission for rare leukaemic cells which might indicate residual disease and/or early signs of relapse. The results indicate that leukaemic cells are demonstrable in remission patients. Analysis of cell populations with antileukaemic sera may therefore have important diagnostic and prognostic potential.

REFERENCES

Baker, M. A., Ramachandar, K. & Taub, R. N. (1975) Specificity of Heteroantisera to Human Acute Leukaemia Associated Antigens. *J. clin. Invest.* (in press).

Brown, G., Capellaro, D. & Greaves, M. F. (1975) Leukaemia Associated Antigens in Man. *J. exp. Med.* (in press).

Brown, G., Greaves, M. F., Lister, A., Rapson, N. & Papamichael, M. (1974) Expression of Human T and B Lymphocyte Cell Surface Markers on Leukaemia Cells. *Lancet*, ii, 753.

Greaves, M. F., Brown, G., Rapson, N. & Lister, T. A. (1975) Antisera to Acute Lymphoblastic Leukaemia Cells. I. An Analysis of Cellular Specificity. *Immunol. clin. Immunopath.* (in press).

Greaves, M. F., Owen, J. T. & Raff, M. C. (1973) T and B Lymphocytes: their Origins, Properties and Roles in Immune Responses. Amsterdam: Excerpta Medica.

Harris, R. (1973) Leukaemia Antigens and Immunity in Man. *Nature, Lond.*, **241**, 95.

Monhanakumar, T., Metzgar, R. S. & Miller, D. S. (1974) Human Leukemia Cell Antigens: Serological Characterization with Xenoantisera. *J. natn. Cancer Inst.*, **52**, 1435.

Pegrum, G. D. (1973) Leukaemia Antigens. *J. Haemat.*, **24**, 1.

Seligmann, M., Preud'homme, J. L. & Brouet, J. C. (1973) *Transplantn Rev.*, **16**, 85.